# Nutritional Management in Chronic Pancreatitis: From Exocrine Pancreatic Insufficiency to Precision Therapy

**DOI:** 10.3390/nu17172720

**Published:** 2025-08-22

**Authors:** Angelo Bruni, Luigi Colecchia, Giuseppe Dell’Anna, Davide Scalvini, Francesco Vito Mandarino, Andrea Lisotti, Lorenzo Fuccio, Paolo Cecinato, Giovanni Marasco, Gianfranco Donatelli, Giovanni Barbara, Leonardo Henry Eusebi

**Affiliations:** 1 Gastroenterology Unit, IRCCS Azienda Ospedaliero-Universitaria di Bologna, 40138 Bologna, Italy; angelo.bruni4@unibo.it (A.B.); luigi.colecchia@studio.unibo.it (L.C.); lorenzo.fuccio3@unibo.it (L.F.); paolo.cecinato@aosp.bo.it (P.C.); giovanni.barbara@unibo.it (G.B.); 2 Department of Medical and Surgical Sciences, University of Bologna, 40138 Bologna, Italy; giovanni.marasco4@unibo.it; 3 Gastroenterology and Gastrointestinal Endoscopy Unit, IRCCS San Raffaele Hospital, Via Olgettina 60, 20132 Milan, Italy; dellanna.giuseppe@hsr.it (G.D.); mandarino.francesco@hsr.it (F.V.M.); 4 Gastroenterology and Gastrointestinal Endoscopy Unit, IRCCS Policlinico San Donato, Piazza Edmondo Malan 2, 20097 San Donato Milanese, Italy; 5 Unité d’Endoscopie Interventionnelle, Hôpital Privé des Peupliers, 75013 Paris, France; gianfranco.donatelli@unina.it; 6 Pavia-Gastroenterology and Digestive Endoscopy Unit, Department of Internal Medicine and Therapeutics, Fondazione IRCCS Policlinico San Matteo, University of Pavia, 27100 Pavia, Italy; davide.scalvini01@universitadipavia.it; 7 Gastroenterology Unit, Hospital of Imola, University of Bologna, 40026 Bologna, Italy; lisotti.andrea@gmail.com; 8 Department of Clinical Medicine and Surgery, University of Naples “Federico II”, 80138 Naples, Italy

**Keywords:** exocrine pancreatic insufficiency, chronic pancreatitis, pancreatic enzyme replacement therapy

## Abstract

Chronic pancreatitis (CP) precipitates complex malnutrition through synergistic mechanisms: exocrine pancreatic insufficiency–driven maldigestion, duodenal or pancreatobiliary strictures limiting nutrient flow, cholestasis impairing micelle formation, alcohol-related anorexia, pain-induced hypophagia, proteolytic catabolism from type 3c diabetes, and a chronic inflammatory milieu that accelerates sarcopenia and bone demineralisation. Consequent calorie–protein depletion, micronutrient and fat-soluble vitamin deficits, and metabolic derangements markedly amplify morbidity. Pancreatic enzyme replacement therapy (PERT) with targeted micronutrient repletion is foundational; high-protein regimens co-administered with PERT curb muscle loss, and medium-chain triglycerides (MCTs) can augment caloric delivery by bypassing lipase dependence, although their benefit over personalised dietetic counselling is marginal. Optimal dietary fat thresholds and timing of escalation from oral to enteral or parenteral feeding remain unresolved. Comprehensive care also demands alcohol abstinence, effective analgesia and stringent glycaemic control. Serial monitoring—biochemical indices, densitometry, dual-energy X-ray absorptiometry and imaging-based body-composition metrics—permits early detection of high-risk patients and precision tailoring of interventions. Intensified multidisciplinary programmes already improve prognostic endpoints and are unveiling biomarkers of nutritional resilience. A structured, evidence-based strategy integrating PERT, macronutrient engineering, micronutrient repletion and metabolic surveillance is essential to mitigate nutrition-related morbidity, enhance long-term outcomes and optimise quality of life in CP.

## 1. Introduction

Chronic pancreatitis (CP) is now framed as a progressive fibro-inflammatory syndrome of the pancreas driven by a multifactorial interplay between toxic-metabolic, environmental, and genetic factors (Table 1). Repeated acinar-ductal injury provokes irreversible parenchymal fibrosis and calcification, ductal distortion, and eventual loss of both exocrine and endocrine function, with pain and complex morphological sequelae. Among its systemic manifestations, malnutrition is both a hallmark and an accelerator of disease progression. Exocrine pancreatic insufficiency (EPI) precipitates steatorrhea and proteolytic failure, while chronic inflammation, hypercatabolism and reduced oral intake synergistically induce calorie–protein malnutrition, sarcopenia, and specific micronutrient deficits [1]. Studies applying the Global Leadership Initiative on Malnutrition (GLIM) diagnostic criteria report that approximately half of CP cohorts are affected by malnutrition, highlighting its widespread clinical impact [2]. Sarcopenia has emerged as a particularly prevalent and prognostically relevant complication. A 2025 meta-analysis that pooled 16 studies (1398 patients) calculated a sarcopenia prevalence of 39% and a two-fold higher risk versus matched controls [3,4]. Body-composition loss coexists with skeletal consequences: pooled data show osteoporosis in 24% and osteopathy (osteopenia + osteoporosis) in 65% of patients. Underweight status, present in about one-quarter of outpatients, independently correlates with reduced quality of life [5].

Therapeutically, pancreatic enzyme replacement therapy (PERT) remains the pillar for restoring intraluminal digestion and mitigating fat-soluble vitamin depletion. Guideline-driven studies demonstrate that adequate dosing of enteric-coated pancrelipase normalises the coefficient of fat absorption and improves biochemical nutrition markers. Contemporary nutrition protocols now integrate high-protein (≥1.2 g kg^−1^ day^−1^) feeding, targeted repletion of vitamins A, D, E and K, calcium and trace elements, and consideration of medium-chain triglycerides for selected steatorrhoea-dominant phenotypes [6,7]. Advances in computed tomography (CT)- and dual-energy X-ray absorptiometry (DXA)-based body-composition analysis, serum metabolomic panels and faecal elastase-1 profiling permit earlier recognition of patients at nutritional risk and pave the way for “precision nutrition” algorithms that individualise caloric targets, enzyme posology and microbiota-modulating adjuvants [8]. The overarching aim is to blunt malnutrition-related morbidity, preserve musculoskeletal integrity and ultimately improve survival and life quality in this chronically debilitated population.

Globally, CP affects approximately 10 individuals per 100,000 person-years, with a stable male predominance (male-to-female ratio ≈ 2:1) and epidemiological curves that have trended upwards over the past quinquennium [9]. In the United States, contemporary estimates place the annual incidence between 5 and 8 per 100,000 and the prevalence at 42–73 per 100,000; in 2018, the disorder generated more than 37,000 emergency-department visits and held the second-highest 30-day readmission rate among digestive diseases (27%), each readmission costing in excess of 27,000 USD [10].

EPI develops in roughly 20% of patients within five years of diagnosis and approaches 70% after two decades, closely paralleling cumulative parenchymal loss and nicotine exposure [11]. Endocrine failure follows a similar temporal gradient; up to one-half of individuals develop pancreatogenic (type 3c) diabetes within ten years, characterised by brittle glycaemic profiles and amplified micro- and macrovascular risk [12]. These endocrine–exocrine derangements underpin the high burden of malnutrition: underweight BMI occurs in 26% of outpatients, and GLIM-defined malnutrition, as noted, afflicts about 50% of cohorts, with direct repercussions on hospitalisation and surgical outcomes.

Registry data indicate that chronic pancreatitis carries a 4- to 5-fold excess mortality relative to the general population, and the onset of exocrine pancreatic insufficiency further doubles that risk [8]. Alongside persistent pain, patients experience fatigue, muscle weakness, diarrhoea and depression, reported in up to 40%, each undermining dietary intake and metabolic stability. These complications drive frequent, protracted hospitalisations and inflate healthcare costs, whereas rigorous enzyme replacement, high-protein nutritional support and systematic micronutrient monitoring demonstrably cut readmissions, preserve bone mineral density and lower fracture incidence [13]. To synthesise current evidence, we performed a narrative, evidence-based review based on a structured search through May 2025, focusing only on adult cohorts and excluding paediatric populations because their growth-related nutritional requirements, diagnostic pathways and weight-based PERT dosing differ substantially. We summarise diagnostic standards for EPI and comprehensive nutritional assessment, emphasising GLIM-based phenotyping, body-composition metrics and bone-health evaluation, and translate these into practice with dietary prescriptions, protein–energy targets and laboratory-guided micronutrient repletion. We delineate PERT formulations, dosing and titration with explicit response and failure criteria; outline escalation from structured counselling to oral nutritional supplements and enteral nutrition, reserving parenteral support for selected indications; and address adjuncts—acid suppression, diagnosis and treatment of SIBO and microbiota-directed strategies—with specific considerations for altered postoperative anatomy and type 3c diabetes; finally, we map precision-nutrition pathways integrating FE-1 stratification, imaging-derived body composition and metabolomic profiling.

## 2. Pathophysiology of Malnutrition in Chronic Pancreatitis

Malnutrition is a common complication in CP: it arises from multiple overlapping factors, including reduced oral food intake caused by chronic abdominal pain and nausea/vomiting, exocrine and endocrine insufficiency, gastrointestinal dysmotility, diarrhoea, chronic inflammation, and gut microbiota alterations [14]. These disturbances combine to limit dietary intake and nutrient processing.

Reduced secretion of pancreatic enzymes and bicarbonates impairs the digestion of both macronutrients and micronutrients [12]. Triglyceride digestion is particularly affected: lipid maldigestion leads to wasting and deficiency of fat-soluble vitamins A, D, E and K [9]. Protease deficiency in EPI also causes protein-energy malnutrition and can impair vitamin B12 absorption [9]. This is aggravated by systemic inflammation, since recurrent pancreatic injury leads to the release of pro-inflammatory cytokines (e.g., TNF-α, IL-6) and acute-phase proteins, sustaining a continuous catabolic state, anorexia, increased basal energy expenditure, and proteolysis [8]. Chronic inflammation also disrupts micronutrient metabolism, accelerating bone resorption and depleting vitamin D stores [15].

A further destabilising event, usually in later phases, is endocrine insufficiency due to the loss of Langerhans islet cells, often referred to as pancreatogenic or type 3c diabetes [16]. This form of diabetes features deficient insulin and often deficient glucagon, causing weak glycaemic control and hypoglycaemia [17]. The lack of insulin shifts metabolism toward gluconeogenesis and further protein lysis, perpetuating catabolism. Glycaemic instability itself may also reduce appetite and increase energy expenditure. Moreover, recurrent postprandial pain and nausea or vomiting, often cause patients to restrict meal size or even skip meals, aggravating calorie deficit [18].

Chronic pancreatitis is also associated with gastrointestinal motility disturbances [19]. Chronic pain, type 3c diabetes, and opioid analgesics contribute to gastroparesis and delayed intestinal transit. These motility defects, along with reduced pancreatic synthesis of antimicrobial peptides and reduced chyme alkalisation favour small intestinal bacterial overgrowth (SIBO) [20,21]. A meta-analysis of nine studies including 336 CP patients found a pooled prevalence of SIBO, mainly diagnosed with glucose or lactulose breath tests, of 36% [95% confidence interval (CI): 17–60%, with considerable heterogeneity (I^2^ 91%)]. Small intestinal bacterial overgrowth worsens malnutrition by consuming nutrients and damaging the brush border, impairing absorption [22].

Refs. [23,24,25] the combined effects of endocrine and exocrine insufficiency, dysmotility, SIBO, chronic inflammation and reduced oral food intake lead to profound nutrient deficiencies. Clinically, vitamin deficiencies can contribute to severe scenarios such as osteopenia/osteoporosis, coagulopathy, night blindness, peripheral neuropathy, and other complications [6]. In a recent meta-analysis on 17 studies involving 1659 subjects with CP, the pooled rate of osteoporosis was 18% (95% CI: 12–23%; *p* < 0.001; I^2^ = 86.3%), and the pooled rate of osteopenia was 39% (95% CI: 31–48%; *p* < 0.001; I^2^ = 91.53%), likely due to poor vitamin D/calcium absorption and chronic inflammation limiting bone anabolism [26,27]. Finally, persistent steatorrhea and weight loss despite enzyme therapy can cause severe malnutrition and sarcopenia, reported in ~39% of patients with CP in a recent meta-analysis on 6 studies with 1556 participants [4]. In this context, autoimmune pancreatitis (AIP) merits separate consideration. Type 1 belongs to IgG4-related disease, and type 2 is defined by granulocytic epithelial lesions with a typical absence of systemic IgG4 involvement. Compared with alcohol-related CP, AIP more often presents with painless obstructive jaundice, cholestasis and extra-pancreatic disease, with frequent IgG4 elevation in type 1 and marked steroid responsiveness [23]. Glucocorticoids are first line; induction with prednisolone can induce rapid clinical, biochemical and radiologic remission and can obviate prolonged biliary stenting, while early therapy limits progression to fixed exocrine and endocrine failure. Given relapse risk and steroid toxicity, a treat-to-target strategy with close monitoring and maintenance in high-risk phenotypes is recommended; B-cell depletion with rituximab is effective but lacks head-to-head randomised controlled trials versus steroids [24]. Similarly to toxic-metabolic forms, EPI and diabetes are frequent at diagnosis, and micronutrient deficits can persist despite pancreatic enzyme replacement therapy. In a cohort of 100 patients, 38% developed at least one deficiency, with zinc in 25.5% and vitamin D in 16.1%, and the odds of any deficiency were increased fivefold in those with exocrine insufficiency (OR 5.1; 95% CI 1.4–18.8); crude associations with relapse attenuated after adjustment [25].

## 3. Diagnosis of Exocrine Pancreatic Insufficiency and Nutritional Assessment

### 3.1. Definition and Pathogenesis

Exocrine pancreatic insufficiency is defined as a decrease in exocrine pancreatic secretion and/or intraluminal pancreatic enzyme activity below the threshold necessary for normal nutrient digestion [9]. Consequently, EPI is associated not only with nutrient malabsorption and deficiencies but also with gastrointestinal symptoms, although clinical manifestations are extremely variable due to the role of several factors [9,28]. Insufficient postprandial stimulation of the exocrine pancreas, along with reduced secretion of pancreatic enzymes and bicarbonate due to pancreatic tissue damage, represent the two primary mechanisms underlying EPI [29,30].

The pathogenesis of EPI in CP is multifactorial. First, parenchymal atrophy and chronic inflammation result in reduced pancreatic enzyme production. Second, ductal obstruction due to intraductal stones and/or strictures compromises the delivery of enzymes to the duodenal lumen. Third, in patients with genetic forms of CP, alterations in the functional activity of pancreatic enzymes may also play a role. Fourth, altered upper gastrointestinal anatomy following resective or bypass pancreatic surgery may disrupt the coordination between chyme transit and pancreatic enzyme secretion, further contributing to maldigestion [31].

### 3.2. Epidemiology

The lifetime risk of developing EPI in patients with CP is high, with a reported prevalence ranging from 20% to 90%. Data from the Scandinavian Baltic Pancreatic Club (SBPC) database, published in 2017, including 910 CP patients, mostly alcoholic in aetiology, showed a prevalence of EPI of 68%, [32]. with other datasets reporting a prevalence of EPI in 60–90% of patients within ten years of CP diagnosis, with toxic aetiologies, such as chronic alcohol consumption and tobacco use, identified as significant risk factors [32].

This variability is closely related to the duration, aetiology, and severity of the disease [33,34]. A recent study from the Dutch Chronic Pancreatitis Registry, which included 987 patients with CP and a median disease duration of 83 months (interquartile range (IQR) 41–158), demonstrated that patients with EPI had significantly longer disease duration [33]. Specifically, the median duration was 26 months longer in patients with definite EPI compared to those without EPI (*p* < 0.001). An additional noteworthy finding from the study by Kempeneers et al. is the progressive increase in EPI prevalence over time, rising from 20% after 5 years of disease to 70% after 20 years [33]. Additionally, CP aetiology plays a crucial role in the timing of development and severity of EPI. The EPI development is significantly more rapid in exotoxic CP patients (mean 13.1 years) compared to genetic and idiopathic CP (mean 26.3 years) [35]. Lastly, the presence of intraductal calcifications was recognised as a significant risk factor for EPI, consequentially increasing the rates of malnutrition [36].

### 3.3. Diagnosis

The diagnosis of EPI requires an integrated approach. Commonly available pancreatic function tests, clinical symptoms, and nutritional deficiencies each have limited specificity. Therefore, EPI should not be diagnosed based on any single diagnostic parameter. Instead, a combined assessment is necessary in each relevant clinical context to ensure accurate diagnosis (Table 2) [37,38].

First, clinical evaluation is fundamental. Patients with a history of chronic pancreatitis or pancreatic head cancer, or those who have undergone total pancreatectomy or pancreaticoduodenectomy, and who present with symptoms of malabsorption, have an extremely high pre-test probability of EPI [29,39,40].

Clinical evaluation includes the examination of symptoms and signs linked to the malabsorption of fat-soluble vitamins (A-D-E-K). These nonspecific manifestations include fatigue, steatorrhea, bloating/abdominal pain, osteoporosis, change in iron metabolism, ecchymosis, impaired night vision, xerophthalmia, ataxia, peripheral neuropathy, and muscle spasms [28,31,41]. In patients with known pancreatic disease, clinically evident steatorrhea and flatulence represent the two most common symptoms, with a frequency that ranges from 15% to 70% and from 55% to 100%, respectively [42].

### 3.4. Nutritional Assessment

Together with clinical evaluation, nutritional assessment plays a pivotal role in the EPI diagnosis in patients with CP. At the time of diagnosis, many CP patients already present with weight loss, often resulting from chronic inflammation, EPI, and associated comorbidities [43]. Although screening tools such as the Malnutrition Universal Screening Tool (MUST) and the Nutritional Risk Score (NRS-2002) are commonly recommended, they were not specifically developed for CP and may have limitations in predicting clinically relevant outcomes [28,43]. For instance, MUST demonstrates high specificity (80%) but low sensitivity (34%) in forecasting hospitalisation risk [2,43]. More recently, the GLIM has proposed a more comprehensive framework for nutritional assessment, integrating phenotypic criteria (e.g., unintentional weight loss, low BMI, reduced muscle mass) with etiologic factors such as inflammation or malabsorption [44,45].

Another fundamental aspect of a correct nutritional assessment is the evaluation of sarcopenia. CT is frequently used to assess muscle mass in CP patients and has suggested a sarcopenia prevalence of approximately 30–40%, though standardised thresholds and functional correlates remain unclear [46]. Alternatively, bioelectrical impedance analysis (BIA) offers a practical and noninvasive method for evaluating body composition. In particular, the phase angle (PhA), derived from BIA, has shown prognostic value across several chronic diseases and correlates with muscle status and inflammatory burden [47]. However, data on its application in CP is still limited. In their study, Olesen et al. evaluated 184 patients with chronic pancreatitis using BIA. Sarcopenia was identified in 17% of cases, the majority of whom had a normal BMI [48]. In a more recent multicentre study, Wiese et al. assessed 66 CP patients using both BIA and handgrip strength measurements. Over 60% of patients showed signs of malnutrition; however, handgrip strength was not associated with malnutrition but only with muscle mass. The proportion of patients presenting both reduced muscle mass and decreased handgrip strength was limited to just 3% [45].

Adequate bone health checks are critical during patients’ evaluations. In fact, bone disease, including osteopenia and osteoporosis, is a frequent and often underrecognised complication [9,28,49]. Several patient-related factors have been associated with low bone mineral density (BMD), including advanced age, female sex, smoking, alcohol consumption, low BMI, and nutritional deficiencies. Fat-soluble vitamin deficiencies, especially vitamins D and K, are common in CP and play a crucial role in bone metabolism, linking EPI and malabsorption to skeletal fragility. Accordingly, the fracture risk in CP is significantly increased, reaching 6% in some cohorts, with a predominance of low-impact fractures involving the hip, vertebral bodies, and distal radius [49]. Despite the clinical relevance, only about one in four patients undergoes appropriate bone health screening, suggesting that bone complications remain an underestimated source of morbidity [49].

However, data comparing vitamin D levels between CP patients and healthy controls are inconsistent. Hoogenboom et al. and Duggan et al. both reported high rates of vitamin D deficiency in CP, but without significant differences from control groups [50,51]. Conversely, another study found that a significantly larger proportion of CP patients had serum vitamin D levels below 30 ng/mL compared to controls [52]. Inconsistencies across studies may stem from heterogeneity in study populations, lack of standardisation in laboratory assays, seasonal variation in sun exposure, and the role of vitamin D as a negative acute-phase reactant [53].

Considering these findings, guidelines recommend baseline assessment of fat-soluble vitamin levels and BMD via DXA in all CP patients, with repeat evaluations every two years in those with osteopenia [8,28,54]. Referral to a bone specialist is advised in cases of osteoporosis for appropriate pharmacologic intervention. Nonetheless, real-world adherence to these recommendations remains low, and high-quality evidence on the impact of PERT or bone-specific therapies on fracture prevention in CP is still lacking [55].

Alternative testing tools are under investigation. A pilot study by Baltar et al. proposed the use of opportunistic CT scan assessment as a viable method for osteoporosis screening in CP patients, especially when CT imaging is already available for other indications [56]. Biochemical markers of bone turnover also appear altered in CP. In one study, levels of procollagen type I N-terminal propeptide (PINP) and osteocalcin were higher in CP patients compared to controls. Patients with vitamin D levels below 20 ng/mL showed significantly increased PINP concentrations, suggesting an imbalance in bone remodelling [52].

### 3.5. Pancreatic Function Tests

A range of diagnostic modalities is available to assess exocrine pancreatic function, although each has specific limitations in terms of accuracy, availability, or practicality [57]. Direct pancreatic function tests—such as the aspiration of duodenal fluid following hormonal stimulation with secretin, cholecystokinin, or cerulein—provide detailed information on pancreatic secretory capacity. However, these procedures are invasive, time-consuming, and primarily reserved for research settings or specialised centres due to their complexity and low accessibility [31].

Among the indirect methods, the quantitative measurement of faecal fat with calculation of the coefficient of fat absorption (CFA) has traditionally been regarded as a gold standard for assessing steatorrhea. This test involves adherence to a high-fat diet followed by a 72 h stool collection, making it cumbersome and largely impractical in routine clinical care. The 13C-mixed triglyceride breath test offers a less invasive alternative by quantifying labelled carbon dioxide in exhaled air after ingestion of a 13C-labelled triglyceride substrate. It reflects the combined processes of lipolysis, absorption, and oxidation of dietary fat, and it has the additional benefit of allowing for dynamic monitoring of PERT efficacy [58]. Nevertheless, its limited availability and lack of standardisation have restricted its widespread clinical adoption.

In current practice, faecal elastase-1 (FE-1) measurement is the most used non-invasive test for diagnosing EPI [59]. FE-1 is stable in stool and unaffected by pancreatic enzyme supplementation, making it convenient for outpatient use. A threshold of 200 μg/g is widely accepted as the normal cutoff; values between 100 and 200 μg/g are considered indeterminate or suggestive of mild-to-moderate insufficiency, whereas concentrations below 100 μg/g are highly specific for moderate-to-severe EPI. A meta-analysis comparing FE-1 with faecal fat quantification reported a sensitivity of 96% and specificity of 88%, underscoring its diagnostic reliability, particularly for more advanced cases of EPI [38].

A key pitfall of FE-1 testing is that it may not accurately detect mild or moderate exocrine pancreatic insufficiency; thus, FE-1 is not useful for early-stage chronic pancreatitis patients. Moreover, the test results can be affected by stool consistency, so watery or diluted stool samples may lead to inaccurate results and false positive diagnoses of EPI. Finally, patients with upper gastrointestinal anatomy alterations (such as in the case of gastrectomy) could present normal FE-1 values but present with exocrine pancreatic insufficiency.

## 4. Dietary Management

### 4.1. General Dietary Indications

Nutritional goals include preserving body weight and lean mass, preventing micronutrient deficiencies, optimising glycaemic control, and minimising pain-related eating restrictions [1,8]. According to the international guidelines, patients with CP without nutritional deficits should follow a balanced diet and do not need to follow a restrictive diet [1,8,60,61,62]. It should be noted, however, that no clinical trials are available comparing the short- and long-term outcomes of dietary changes versus a balanced diet in this specific setting; therefore, the quality of evidence is low.

Strict long-term fat restriction is no longer universally advocated. There is no high-quality evidence that a very-low-fat diet improves pancreatitis outcomes, and unnecessary restriction can worsen an already fragile nutritional status [1,63,64]. Nonetheless, there is little consensus on the management of diet in patients without nutritional deficits in whom significant pain is present after meals. In fact, abdominal pain in CP is often triggered by food intake that stimulates pancreatic secretion. General dietary strategies in this case include a tailored approach: during acute flares or in pain-prone individuals, moderate fat reduction for a limited time may be tried with the aim of reducing excessive cholecystokinin (CCK) release and avoiding pancreatic secretion. In all cases, patients should avoid binge eating or prolonged fasting, and in case of the failure of traditional advice, peptide-based enteral formulas rich in medium-chain triglycerides (MCTs) or even elemental diets may be tried [65,66,67]. Strong consensus is present internationally on the importance of complete abstinence from alcohol and smoking, as they are considered among the most important risk factors and pain triggers in CP.

### 4.2. Dietary Indications for Patients with Malnutrition

General nutritional advice from international guidelines for malnourished patients with CP is to consume high-protein, high-energy food in five to six small meals per day. (Table 3) In order to reduce the risk of hypoglycaemia, especially in patients with type 3c diabetes, patients should avoid skipping meals [1,8]. No specific recommendation is given on the daily dosage of macronutrients [1,8]. However, as patients typically have increased resting energy expenditure of approximately ~30–35 kcal/kg/day to offset hypermetabolism, this could be a reasonable starting point for dietary counselling [63]. In order to reach a high intake of foods with limited fat intake, significant protein intake (~1.2–1.5 g/kg/day; consisting of meat, fish, eggs and legumes) and a high-carbohydrate diet (whole grains and legumes) are advised to support lean tissue [68]. Fat intake may be tolerated at ~25–35% of total calories if pancreatic enzyme replacement is optimised rather than enforcing a strict low-fat diet [69]. In both patients with and without EPI, fibre and meal volume require consideration. Even though the recommendation is based on a few dated studies, a very high-fibre diet may bind nutrients and worsen steatorrhea; therefore, fibre should be kept at a moderate level, especially if enzyme dosing is suboptimal [70].

### 4.3. Micronutrient Supplementation

In CP, intraluminal maldigestion and malabsorption—driven by EPI, an acidic duodenal environment, bile-acid dysmetabolism and small-bowel dysbiosis—produce deficits in fat-soluble vitamins (A, D, E, K), water-soluble vitamins (B12, folate, thiamine) and key minerals such as calcium, magnesium and zinc [51,71,72,73]. Guidelines uniformly recommend routine monitoring and replacement of these nutrients. Patients should have periodic lab assessments of 25-OH vitamin D, vitamins A and E, and coagulation profiles [1,8,60,61,65,74]. Notably, both Japanese and European guidelines advise against blind supplementation of all fat-soluble vitamins for all patients with CPs and suggest supplementing vitamins A, D, E, and K once EPI is present [1,65]. Although water-soluble vitamin deficiency may be present, it should be noted that it is much rarer. In fact, in recent case–control studies between patients with CP and controls, no statistical difference in water-soluble vitamin deficiency was noted [73,75]. Regardless, it is still recommended to routinely test and treat for vitamin B12, folic acid and thiamine deficiency, especially in patients with alcohol use disorder [1,8].

### 4.4. The Role of Multidisciplinary Teams and Psycho-Social Counselling

Further general recommendations on nutrition in CP include patients’ and caregivers’ education on diet, adequate symptom monitoring and aid in quitting alcohol and tobacco. Meal diaries or written dietary regimens might assist patients in monitoring their consumption and symptoms [76]. A pancreatic disease-experienced dietitian who can modify dietary objectives, customise meal planning, and suggest oral supplements should be part of the treatment team [64,77,78].

Lastly, CP is linked to a high disease burden, a substantial detrimental influence on quality of life and elevated rates of anxiety and depression. These conditions can diminish appetite, lead to emotional eating or avoidance of eating due to fear of pain and impair adherence to dietary regimens. For these reasons present-day viewpoints on CP treatment have changed to support a multidisciplinary strategy that provides psychological aid to help people cope with pain and adjust to chronic disease and social instances [79]. Therefore, clinicians should screen for depression and anxiety and recognise their impact on nutrition. Behavioural interventions such as relaxation techniques, mindfulness or group support may reduce pain perception and facilitate normal eating patterns and should be a fundamental aid to analgesic pharmacological therapy [79]. These concepts underscore the need for a multidisciplinary team composed of general practitioners, gastroenterologists, dieticians, endocrinologists, psychiatrists, and psychologists.

## 5. Principles of Pancreatic Enzyme Replacement Therapy

PERT is a cornerstone in the management of CP when there is a concomitant EPI. Its primary goal is to restore adequate digestion, to improve nutrient absorption and decrease gastrointestinal symptoms. As a matter of fact, the maldigestion of lipids is the main cause of malnutrition and underlies key gastrointestinal manifestations occurring during CP, such as steatorrhea, weight loss, bloating, and abdominal discomfort. While the human body has other compensatory mechanisms for metabolisation of proteins and carbohydrates, the pancreas is the only one responsible for lipid digestion, making lipase replacement the central focus of PERT.

PERT is administered orally, mainly through pH-dependent enteric-coated microspheres or capsules that carry amylase, lipase, and protease to compensate for CP enzymatic deficiencies. These formulations are designed to resist gastric acidity and release their enzymatic content in the small intestine at a pH ≥ 5.5. In the case of non-enteric-coated formulations, the administration of a proton pump inhibitor is required to maximise their effectiveness [80]. All currently approved drugs are porcine-derived, and no synthetic or human-derived enzyme formulations are available. These products differ in their enzyme ratios (lipase, amylase, and protease) and inactive ingredients and are labelled according to their lipase activity [81]. To date, there is no head-to-head randomised controlled trial (RCT) comparing the effect of different products for EPI in CP. The only direct comparison comes from studies in cystic fibrosis-related EPI, where no significant differences in CFA were observed between two commonly prescribed formulations [82].

In CP-related EPI, PERT should be taken with every lipid-containing meal or snack, starting with the first bites and repeated mid-meal to optimise intraluminal mixing and lipolysis. Dosing is individualised to meal fat load, EPI severity and gastrointestinal anatomy; a pragmatic adult starting regimen is 40,000–50,000 U lipase with main meals and ~20,000–25,000 U with snacks, with stepwise titration to clinical response—resolution of steatorrhoea, normalisation of stool form and frequency, weight stabilisation, and correction of fat-soluble vitamin deficits [80]. Head-to-head RCTs among pancrelipase formulations in adult CP are lacking; current practice therefore rests on a class effect and limited extrapolation from CF, where approved products show no clinically meaningful differences. In refractory steatorrhoea despite adherence, escalation to 75,000–100,000 U lipase per meal is reasonable, coupled with a structured review of modifiable factors (dietary fat and excess fibre, SIBO and glycaemic instability).

Acid suppression with a PPI can be trialled when low intraduodenal pH is suspected (e.g., rapid gastric emptying, postsurgical anatomy) or when clinical targets are unmet on adequate dosing; however, evidence for an additive effect of PPIs is mixed—small randomised and physiological studies in CF-EPI have not demonstrated consistent improvements in fat absorption Refs. [83,84]—so PPI co-therapy should be time-limited and continued only if predefined endpoints are achieved [85,86,87,88]. In altered postoperative anatomy, microgranular enteric-coated preparations <2 mm that co-empty with chyme, meticulous dose splitting and routine PPI co-therapy are preferred.

The efficacy of PERT is most assessed in clinical trials using CFA as the primary endpoint, and all the PERT bring improvements in CFA levels, stool frequency and quality of life compared to placebo [89]. On the contrary, improvements in nutritional parameters such as fat-soluble vitamins, albumin, prealbumin and haemoglobin yielded inconsistent results across studies. Replacement therapy is generally well tolerated, but a few studies reported adverse events such as nausea and constipation related to higher doses [90].

## 6. Adjuvant and Emerging Therapies

### 6.1. Beyond Standard Nutritional Advice

The majority of patients with CP complicated by malnutrition can be effectively managed with dietary advice and PERT. However, typically 10–15% of patients do not reach preset dietary goals and require further therapy such as oral nutrition supplementation (ONS) or enteral or parenteral feeding. Enteral formulae consisting of pre-digested products and a mixture of long-chain fatty acids (LCFAs) and MCTs have been proposed as therapeutic solutions [64,91]. However, MCTs yield less energy when compared to LCFAs and are often associated with adverse effects like cramps, nausea, and diarrhoea [91]. Up to now, studies have have not demonstrated any clear benefit of MCTs over LCFAs when used in combination with enzyme supplementation or over adequate dietary counselling [76,91].

If oral intake is insufficient or gastrointestinal symptoms prevent adequate nutrition, enteral nutrition (EN) is recommended. Naso-gastric tube-feeding (or naso-jejunal if gastric outlet syndrome symptoms or gastroparesis are present) is generally well tolerated and can improve weight gain and pain control and reduce analgesic requirements [1,6,64,92,93]. If long-term support (>4–6 weeks) is needed, a gastrojejunostomy tube may be placed to reduce patients’ discomfort. Although no comparative studies have been performed on the matter, it is reasonable in these cases to start with standard (polymeric, whole-protein) formulas and to use peptide-based or even elemental formulas in case of intolerance.

The last resort to maintain an adequate daily energy intake in case of failure of all previous therapies or rare situations such as duodenal obstruction, complex fistulae or perioperative nutrition in severely malnourished patients is parenteral nutrition (PN) (<1% of cases) [94]. In the case of PN, central line positioning is recommended along with vigilant control of potential infectious and hepatologic complications.

### 6.2. Gut–Pancreas Axis

The gut–pancreas axis is an emerging target in CP. As previously described, CP patients often have SIBO, which may worsen malnutrition and inflammation and can be a treatment target [21,95,96]. The cornerstone of SIBO’s treatment, once a diagnosis based on symptoms and an indicative hydrogen or lactulose test has been made, is antibiotic therapy [97]. No trials have been performed yet on antibiotics for SIBO in the context of CP; however, recent data show a possible role of probiotics as an adjunctive therapy to antibiotics in SIBO treatment [83]. A relatively recent prospective, randomised, controlled, double-blind clinical trial on 60 patients with CP reported that a mix of probiotic and prebiotic supplements (*Lactobacillus casei*, *Lactobacillus rhamnosus*, *Lactobacillus acidophilus*, *Bifidobacterium bifidum* and fructooligosaccharides) significantly improved laboratory measures in CP patients and decreased the number of patients with diarrhoea [84]. Moreover, another prospective, single-blind, randomised, controlled trial including 75 patients with CP undergoing Frey’s procedure, a type of partial pancreatectomy, showed that perioperative probiotic and prebiotic therapy (*Streptococcus faecalis*, *Clostridium butyricum*, *Bacillus mesentericus*, *Lactobacillus sporogenes* and fructooligosaccharides) markedly reduced postoperative infections and hospital stay [98]. No data is currently available on different ways to modulate microbiota, such as other types of probiotics, postbiotics, functional foods or faecal microbiota transplantation [99].

### 6.3. Genetic Predisposition in Chronic Pancreatitis

Hereditary chronic pancreatitis (HCP) is a very rare form of early-onset chronic pancreatitis [100]. Eighty percent of family members with HCP have recurrent acute pancreatitis leading to chronic pancreatitis due to mutations in the cationic trypsinogen (PRSS1) gene. In addition to HCP, a number of genetic variations, in order of decreasing risk effect in the PRSS1, CPA1, SPINK1, CTRC, CEL and CFTR genes, have been found to raise the risk of developing chronic pancreatitis, particularly when other risk factors are present [101]. Heterozygous or homozygous mutations in these genes accelerate disease onset and severity, and identifying them allows risk stratification and tailored interventions [101]. According to current guidelines, individuals under 20 years of age and those with a family history of two or more first- or second-degree relatives with CP should obtain genetic testing [28]. Outside of research, genetic testing for other genes is not advised, and only a sweat test should be carried out to rule out cystic fibrosis in adults with idiopathic CP who do not exhibit any additional clinical symptoms of the disease [28]. Out of the abovementioned genes, the only targetable one is CFTR (cystic fibrosis transmembrane conductance regulator), which impairs ductal ion transport, yielding a higher CP risk. Early use (between 12 and 24 months) of CFTR modulators in cystic fibrosis may allow the recovery of pancreatic function, which on one hand may reduce the detrimental effects of severe EPI, but on the other may increase the number of CP recurrences due to the existence of remaining acinar cells which can cause new attacks [102,103].

## 7. Conclusions and Future Perspectives

CP imposes a persistent catabolic state that, combined with both exocrine and endocrine insufficiency, contributes to complex malnutrition, progressive sarcopenia, metabolic bone disease, and micronutrient deficiencies (Table 4). Early identification through a combined phenotypic and etiologic evaluation, followed by timely initiation of pancreatic enzyme replacement therapy, is essential to restore effective intraluminal digestion and to avert deficiencies in fat-soluble vitamins.

High-protein feeding (≥1.2 g kg^−1^ day^−1^), judicious fat provision assisted by adequate lipase dosing, and systematic repletion of vitamins A, D, E, K, calcium and trace elements mitigate muscle wasting, bone loss and immune compromise. Multidisciplinary programmes integrating dietetics, endocrinology, analgesia and psychosocial support demonstrably reduce hospitalisations and improve quality of life.

Looking into the future, precision-nutrition algorithms that combine faecal elastase-1 stratification, metabolomic profiling and imaging-based body composition may allow enzyme and nutrient prescriptions to be titrated in real time. Randomised trials are needed to define the ideal lipid fraction, clarify the role of probiotics and evaluate the long-term skeletal impact of early CFTR modulation.

## Figures and Tables

**Table 1:** Multifactorial drivers of malnutrition in chronic pancreatitis.

Reduced Oral Intake	Maldigestion E Malabsorption	Hypercatabolism E Metabolic Dysfunctions
Postprandial abdominal pain Nausea and vomiting Early satiety and bloating Neuro-hormonal anorexia (CCK, GLP-1, leptin) Depression or anxiety interfering with diet Voluntary food restriction to avoid pain Diabetic gastroparesis	Exocrine pancreatic insufficiency Acidic duodenal pH → bile-salt precipitation Steatorrhoea with loss of ADEK vitamins Small-intestinal bacterial overgrowth (SIBO) and dysbiosis Biliary or duodenal strictures causing stasis Dilated or obstructed pancreatic duct with stones	Chronic low-grade inflammation (IL-6, TNF-α) Pancreatogenic diabetes with insulin and glucagon deficiency → proteolysis Oxidative stress and systemic catabolism Accelerated skeletal-muscle breakdown → sarcopenia Micronutrient-driven endocrine disruption (vitamins D and K)

**Table 2:** Laboratory and radiologic signatures of chronic pancreatitis.

Biochemical Markers	Typical Imaging Findings
↓ Faecal elastase-1 ↓ Serum trypsinogen ↓ Fat-soluble vitamins A, D, E, K ↓ 25-OH-vitamin D ± ↓ Ca^2+^ & ↑ PTH ↓ Albumin, pre-albumin ↓ Mg, Zn, Se ↓ Vitamin B12 ↑ Homocysteine ↑ HbA1c / fasting glucose ↓ C-peptide (type 3c DM) ↑ CRP, ESR; ↑ IL-6, TNF-α ↓ Phase angle on BIA	CT/MRI/MRCP → duct “chain-of-lakes”, coarse calcifications, gland atrophy, pseudocysts/WON CE-CT/CTA → vascular events (splenic- or portal-vein thrombosis, pseudoaneurysm) EUS → hyperechoic foci and strands, lobularity, duct wall thickening and alternating strictures/sacculations, intraductal stones, cysts Ultrasound → ductal dilatation, parenchymal calcifications, cystic lesions DXA → low BMD CT body composition/MRI-PDFF → visceral fat loss, ↓ skeletal muscle area

Symbols: ↓ = decreased/low; ↑ = increased/high;

**Table 3:** General dietary recommendations in chronic pancreatitis with nutritional deficits.

Dietary Aspects	Recommendations
Caloric intake	High-energy intake (~30–35 kcal/kg) to be adjusted over time. Monitor intake via food records. Best if dietary counselling is present.
Protein	Aim for ~1.2–1.5 g/kg/day to preserve lean mass.
Fat intake	~25–35% of calories from fat if PERT is effective. Fat-free diets are not recommended.
Carbohydrates	Complex carbohydrates are preferred over simple sugars to maximise energy intake. Special attention to glycaemic control in type 3c diabetes.
Fibres	Avoid very high-fibre foods.
Meal pattern	Small, frequent meals (5–6 per day) and Avoid large-volume meals. Avoid skipping meals to avoid hypoglycaemia.
Pain triggers	Avoid personal trigger foods. Strict alcohol and tobacco avoidance.
Micronutrients	Regularly screen for and supplement deficiencies: vitamins A/D/E/K, calcium, magnesium, and zinc. Do not supplement vitamins blindly. Also screen for water-soluble vitamins and supplement if deficient.
Multidisciplinary team	It is best to create a multidisciplinary team to treat the nutritional deficiencies, especially in severe cases. Assess and address psychosocial problems. Address substance abuse and dietary adherence barriers.

**Table 4:** Summary of key studies on the role and management of malnutrition in chronic pancreatitis of the last 10 years.

Study (Author, Year)	Study Type	Population	Sample Size	Key Findings	Clinical Implication
Rammohan et al., 2015 [98]	RCT	CP patients undergoing surgery	75 CP patients	Probiotics reduced postoperative infections and shortened hospital stays (effect estimates not extracted here).	Consider targeted perioperative microbiome modulation to reduce postoperative morbidity after pancreatic drainage procedures.
Hoogenboom et al., 2016 [50]	Systematic review and meta-analysis	CP patients and healthy controls (HC)	9 studies (465 CP, 378 HC)	High prevalence of vitamin D deficiency/insufficiency in CP, not clearly higher than HC across pooled studies.	Routine screening for hypovitaminosis D in CP is justified even if excess risk vs. HC is inconsistent.
Martínez-Moneo et al., 2016 [72]	Systematic review and meta-analysis	CP patients	12 studies (548 CP)	Fat-soluble vitamin deficiencies are common in CP.	Systematic assessment and replacement of fat-soluble vitamins should be embedded in CP care pathways.
Olesen et al., 2017 [32]	Cohort prospective	CP international registry	910 CP patients	High prevalence of EPI and pancreatogenic diabetes (type 3c) within registry	Systematic screening for EPI and type 3c diabetes is warranted in CP.
Dos Santos et al., 2017 [84]	RCT	CP patients	60 CP patients	Symbiotics improved clinical symptoms and laboratory indices versus control.	Adjunctive symbiotics may benefit selected CP patients with EPI.
Vanga et al., 2018 [38]	Systematic review and meta-analysis	CP patients with and without EPI	9 studies (1101 patients)	FE-1 demonstrated high sensitivity and specificity for moderate–severe EPI compared with the secretin test.	FE-1 is an appropriate first-line, non-invasive test for clinically significant EPI.
Olesen et al., 2019 [48]	Prospective cohort	CP patients	182 CP patients	Sarcopenia occurred even with normal BMI and predicted hospitalisations and lower survival.	Body composition assessment adds prognostic information beyond BMI in CP.
Kempeneers et al., 2020 [33]	Cohort prospective	CP national registry	987 CP patients	EPI prevalence rose from ~20% at 5 years to ~70% at 20 years; alcoholic aetiology is associated with higher risk.	Longer disease duration and alcoholic CP identify patients at high risk for EPI.
Phillips et al., 2022 [43]	Systematic review	CP patients	8 studies (420 CP patients)	Oral nutrition support and counselling improved BMI and overall nutritional status.	Structured nutrition programmes confer measurable benefit and should be implemented.
Gopi et al., 2022 [44]	Retrospective cohort study	CP patients	297 CP patients	Approximately half of CP patients met GLIM criteria for malnutrition.	Applying GLIM systematically identifies a high burden of malnutrition in CP.
Vujasinovic et al., 2023 [25]	Retrospective cohort study	Autoimmune CP patients	100 AIP patients	Micronutrient deficiencies remained common despite PERT.	PERT alone may be insufficient; targeted micronutrient monitoring/repletion is required.
Ramai et al., 2023 [49]	Systematic review and meta-analysis	CP patients	17 studies (1659 CP)	Osteoporosis/osteopenia are prevalent and underscreened in CP.	Bone health assessment should be incorporated into routine CP care.
Khurmatullina et al., 2025 [4]	Systematic review and meta-analysis	CP patients vs. HC	16 studies (1556 CP)	Sarcopenia prevalence is significantly higher in CP than in controls.	High sarcopenia burden supports routine body composition and functional assessment.
Chu et al., 2025 [89]	Systematic review	CP with EPI	28 studies	PERT improves fat absorption and stool quality; evidence is insufficient to compare specific formulations.	PERT is efficacious for EPI in CP; formulation selection can be individualised pending comparative data.

CP Chronic Pancreatitis; RCT Randomised Controlled Trial; HC Healthy Controls; EPI = Exocrine Pancreatic Insufficiency; BMI = Body Mass Index; GLIM = Global Leadership Initiative on Malnutrition; PERT = Pancreatic Enzyme Replacement Therapy; AIP = Autoimmune Pancreatitis.

## Abbreviations

The following abbreviations are used in this manuscript:

ADEK	Fat-soluble vitamins A, D, E and K
BIA	Bioelectrical impedance analysis
BMD	Bone mineral density
BMI	Body mass index
CCK	Cholecystokinin
CEL	Carboxyl-ester lipase gene
CFA	Coefficient of fat absorption
CFTR	Cystic fibrosis transmembrane conductance regulator
CPA1	Carboxypeptidase A1 gene
CP	Chronic pancreatitis
CRP	C-reactive protein
CTRC	Chymotrypsin C gene
DXA	Dual-energy X-ray absorptiometry
EN	Enteral nutrition
EPI	Exocrine pancreatic insufficiency
FE-1	Faecal elastase-1
GLIM	Global Leadership Initiative on Malnutrition
HCP	Hereditary chronic pancreatitis
IL-6	Interleukin-6
LCFA	Long-chain fatty acid
LD	Linear dichroism
MCT	Medium-chain triglyceride
MUST	Malnutrition Universal Screening Tool
ONS	Oral nutritional supplementation
PERT	Pancreatic enzyme replacement therapy
PINP	Procollagen type I N-terminal propeptide
PN	Parenteral nutrition
PRSS1	Protease serine 1 gene (cationic trypsinogen)
RXR	Retinoid X receptor
SIBO	Small-intestinal bacterial overgrowth
SPINK1	Serine peptidase inhibitor, Kazal type 1 gene
TLA	Three-letter acronym
TNF-α	Tumour necrosis factor-alpha
VDR	Vitamin D receptor
VDREs	Vitamin D response elements
